# The Development and Evaluation of an e-Learning Course That Promotes Digital Health Literacy in School-age Children: Pre-Post Measurement Study

**DOI:** 10.2196/37523

**Published:** 2022-05-16

**Authors:** Lars König, Eugenia Marbach-Breitrück, Anne Engler, Ralf Suhr

**Affiliations:** 1 Stiftung Gesundheitswissen Berlin Germany

**Keywords:** digital education, digital health literacy, digital literacy, e-learning, health education, health information, health literacy, learning material, school, school-age children

## Abstract

**Background:**

Experts agree that the promotion of (digital) health literacy should be an integral part of the school curriculum. However, promoting (digital) health literacy within the German school system is difficult because (digital) health education is not a mandatory school subject in all the German states. Therefore, experts suggest that (digital) health literacy could be addressed as part of the mandatory framework for digital education and digital literacy in schools developed by the German Conference on Education Ministries and Cultural Affairs (Kultusministerkonferenz).

**Objective:**

The goal of this study was to evaluate a newly developed e-learning course that was designed to improve (digital) health literacy in school-age children and concurrently to teach skills specified in the mandatory framework for digital education and digital literacy in schools. It was hypothesized that participants’ health literacy and digital health literacy levels would be higher after completing the e-learning course than they were before doing the course. Furthermore, it was hypothesized that after completing the e-learning course, participants’ subjective and objective knowledge in the domain of (digital) health literacy would be higher than it was before doing the course.

**Methods:**

The pre-post measurement study was conducted online. After participants (N=323) gave their informed consent to participate in the study, they provided demographic information and answered all measures (premeasurement). Following this, participants had 7 days to complete the e-learning course. After finishing the e-learning course, participants answered all the measures again (postmeasurement).

**Results:**

To test the hypotheses, Bayesian paired samples *t* tests (1-sided) were conducted. After completing the e-learning course, participants showed higher health literacy levels. Specifically, they showed higher competency levels in the domains of theoretical knowledge (Bayes factor [BF]_–0_=676,000; δ=–0.316), practical knowledge (BF_–0_=92,300; δ=–0.294), critical thinking (BF_–0_=7.42e+13; δ=–0.482), self-awareness (BF_–0_=11,500,000; δ=–0.345), and citizenship (BF_–0_=266,000; δ=–0.306). Furthermore, participants achieved higher digital health literacy levels. Specifically, they achieved higher competency levels in the domains of information searching (BF_–0_=2.339; δ=–0.135), evaluating reliability (BF_–0_=2.03e+11; δ=–0.434), and determining relevance (BF_–0_=316,000; δ=–0.308). Moreover, participants demonstrated higher subjective (BF_–0_=3.58e+82; δ=–1.515) and objective knowledge (BF_–0_=3.82e+97; δ=–1.758) in the domain of (digital) health literacy.

**Conclusions:**

The newly designed e-learning course provides an easy way for schools and teachers from all German states to integrate (digital) health literacy education into their school curriculums and lessons. The evaluated course is especially attractive because it was designed to improve (digital) health literacy and at the same time to teach skills specified in the mandatory framework for digital education and digital literacy in schools developed by the German Conference on Education Ministries and Cultural Affairs (Kultusministerkonferenz).

## Introduction

Health literacy describes people’s ability to evaluate and apply health information in the context of disease prevention and health promotion [1]. Health literacy is one central component of digital health literacy, which refers to people’s ability to effectively use health information derived from online and electronic sources [2]. In recent years, health literacy has become a major public concern because studies have shown that it is linked to various health outcomes and behaviors [3-7]. People with low health literacy, for example, show poorer dieting habits, are less physically active, smoke more frequently, have more sick leave days, and rely more heavily on the health care system [7]. Furthermore, digital health literacy has become an increasingly important topic because people regularly turn to the internet when searching for health information [8-12]. In such situations, people are often confronted with inaccurate online health information and therefore need the ability to evaluate the trustworthiness of information sources and the credibility of their information [10,13-18]. Teaching adequate evaluation strategies seems especially important because laypeople often base their trustworthiness judgments on factors like the enthusiasm of an information source and their tone of voice [19].

In Germany, health literacy has decreased since 2014 [5,6]. A representative survey published in 2021 found that 58.8% of the German population had low health literacy levels and 75.8% had low digital health literacy levels [6,7]. When searching the internet for health information, it is especially important to decide whether the information is reliable and whether it is written with commercial interests [20]. However, 82.6% of the German population find it difficult or very difficult to decide whether information is reliable and 82% report it as difficult or very difficult to assess whether information is written with commercial interests [7]. Even university students, who represent a well-educated subgroup of the German population, find it difficult to decide whether online health information is reliable and written with commercial interests [21]. Such results may arise because information evaluation strategies are not well enough embedded within the German school system and even preservice teachers have problems adapting to the digitization of the educational system [22]. Data from the Program for International Student Assessment, for example, show that during their entire school experience, only 54.3% of the students were taught how to decide whether to trust information from the internet. Furthermore, only 48.7% of the students were taught how to detect whether information is subjective or biased, and only 45.2% had the capacity to distinguish facts from opinions [23].

Such results are unfortunate because teaching (digital) health literacy to school-age children has the potential to improve various health outcomes later in life [24,25]. Furthermore, schools seem to be an ideal place to promote (digital) health literacy because they can reach almost all children within a society [26-28]. In line with this argumentation, the World Health Organization argues that health literacy should be an integral part of the school curriculum [29]. Furthermore, a recent concept paper from the World Health Organization Regional Office for Europe specifically stresses the importance of addressing health literacy as well as digital health literacy in schools [30]. For Germany, addressing (digital) health literacy in schools seems especially important because German pupils demonstrate particularly low health literacy levels compared to pupils from other European countries [31]. However, promoting (digital) health literacy in schools is difficult because (digital) health education is not a mandatory school subject in all German states. To address this issue, experts suggest that (digital) health literacy could be addressed as part of the mandatory framework for digital education and digital literacy in schools, which was developed by the German Conference on Education Ministries and Cultural Affairs (Kultusministerkonferenz) [32,33].

In line with these suggestions, the independent, nonprofit foundation Stiftung Gesundheitswissen developed the free e-learning platform Gesundweiser.de to teach (digital) health literacy to school-age children. On the platform, visitors can receive information on the topic of (digital) health literacy. Furthermore, they can take part in a free e-learning course. The course was designed to improve (digital) health literacy and at the same time to teach skills specified in the mandatory framework for digital education and digital literacy in schools [32]. Even though there are various reasons why (digital) health literacy should be taught in schools [34], research has shown that many school-based health interventions end after external funding stops [35]. To facilitate the permanent implementation of an intervention, it is important to design interventions that recognize the specific needs of schools and teachers [36]. Therefore, the e-learning course was designed to be applicable in various types of schools and subjects. Since the course is self-explanatory and no active supervision is required, it is especially suitable as a homework exercise that can be completed within a set amount of time. Within the course, participants learn how they can evaluate health information on the internet. The provided material was created by a multiprofessional team, including health and e-learning specialists, and was derived from professional guidelines (eg, Guideline for the Development of Evidence-based Patient Information) [37]. The aim of this study was to test the following hypotheses:

- Hypothesis 1: Participants’ health literacy levels will be higher after completing the e-learning course than they were before completing the e-learning course.
- Hypothesis 2: Participants’ digital health literacy levels will be higher after completing the e-learning course than they were before completing the e-learning course.
- Hypothesis 3: Participants’ subjective knowledge in the domain of (digital) health literacy will be higher after completing the e-learning course than it was before completing the e-learning course.
- Hypothesis 4: Participants’ objective knowledge in the domain of (digital) health literacy will be higher after completing the e-learning course than it was before completing the e-learning course.

## Methods

### Sample

German pupils between the age of 16 and 20 years were recruited from all German states and different types of schools via an online panel provided by a market research company (SPLENDID RESEARCH GmbH). The German school system is highly complex. Within the 16 German states, various types of schools, subjects, and core curriculums exist. To serve a wide range of pupils, the course was not designed for a specific type of school and core curriculum. Instead, it was designed for pupils between the age of 16 and 20 years. Since 16-year-old pupils typically attend the 10th grade, the course can be used in diverse types of schools with a 10th grade (eg, Hauptschulen, Realschulen, Gesamtschulen, Gymnasien). Furthermore, it can be used in higher grades (eg, Gesamtschulen, Gymnasien) and vocational schools (Berufsschulen) as well. As compensation for participating in the study, participants received a 60€ (exchange rate in January 2022: 1€ ≈ USD 1.1342) online shop voucher. An a priori power analysis using G*Power (University of Düsseldorf) indicated that a total of 272 participants was needed to detect a small-to-medium effect with satisfactory power (specifications: test family = *t* tests; statistical test = means: differences between 2 dependent means [matched pairs]; type of power analysis = a priori: compute required sample size – given α, power, and effect size; tail(s)=1; effects size dz=0.2; α err prob=.05; power [1–β err prob]=0.95) [38]. To compensate for possible participant exclusions and data collection problems, it was decided to oversample slightly.

### Ethical Considerations

Before data collection, the study protocol was submitted to the ethics committee of the Berlin Medical Association (Eth-68/21). The ethics committee had no ethical or professional objections to the study protocol.

### Procedure

Data collection took place between December 2021 and January 2022. The pre-post measurement study was conducted online using an online platform for data collection provided by a market research company (SPLENDID RESEARCH GmbH). Prior to the study, participants received detailed information about the context, purpose, and procedures of the study. Furthermore, they were informed that they could opt out of the study at any time. After participants gave their informed consent to participate in the study, they provided demographic information and answered all measures (premeasurement). Following this, participants had 7 days to complete the e-learning course. Because the course was not designed for a specific type of school and subject, it can be used in various contexts. Since the course is self-explanatory and no active supervision is required, it is especially suitable as a homework exercise that can be completed within a set amount of time. To simulate such a homework exercise, participants were given 7 days to complete the course. During this 7-day period, participants could use any device to complete the course and they could start and pause the course as often as they liked. Simulating a homework exercise by giving participants the opportunity to complete the course within 7 days has the advantage that it increases ecological validity. However, it also creates methodological disadvantages that will be discussed in the limitations section.

**Figure 1:**
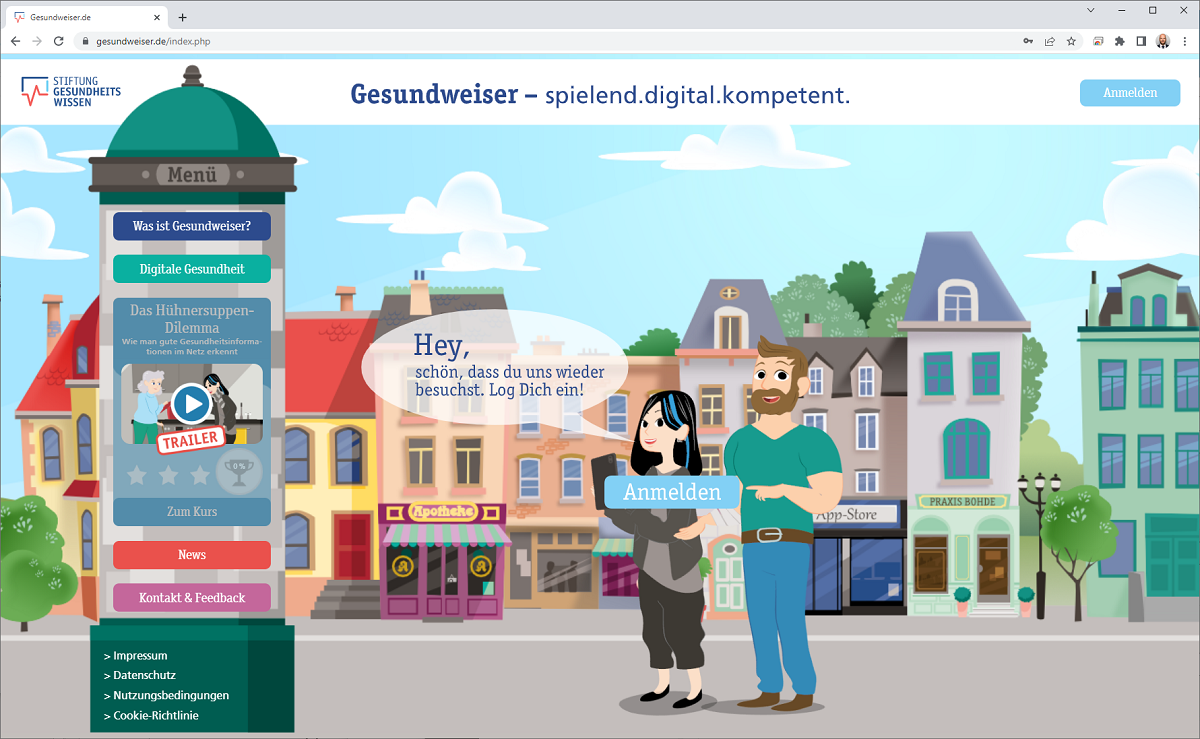
Screenshot of the e-learning platform Gesundweiser.de.

**Figure 2:**
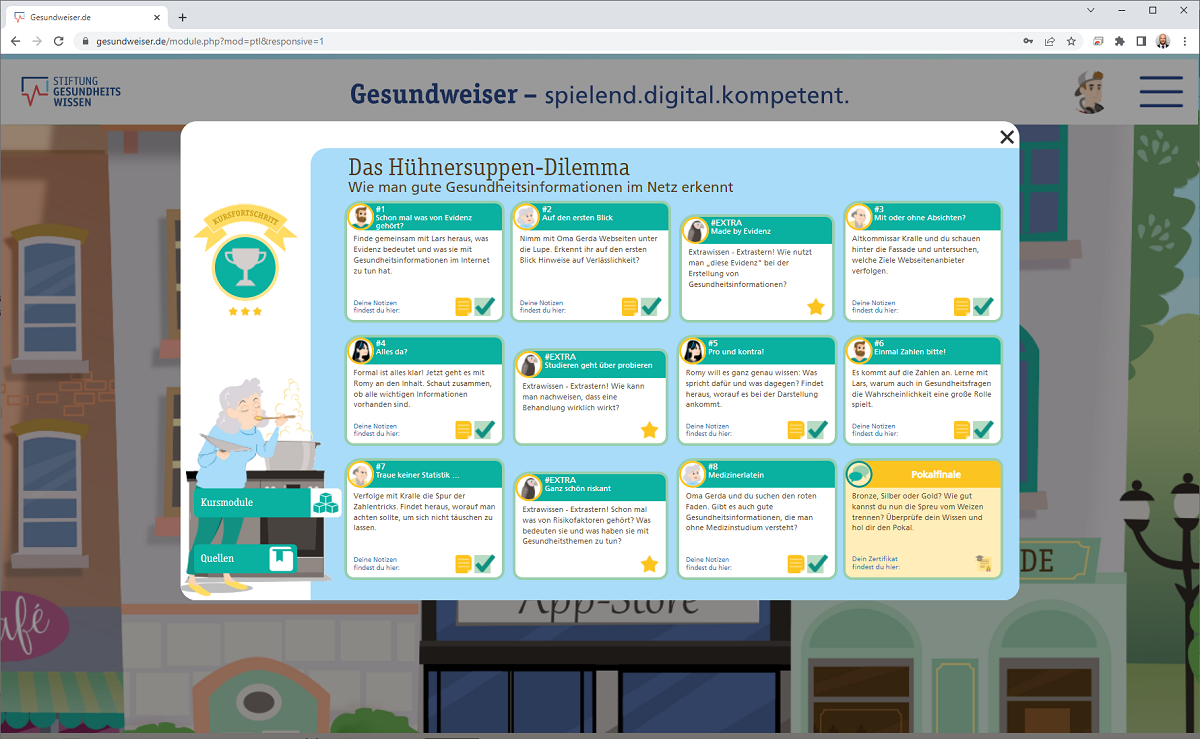
Overview of the e-learning course modules.

Within the course, participant learned how they could evaluate health information on the internet. The provided material was created by a multiprofessional team, including health and e-learning specialists, and was derived from professional guidelines (eg, Guideline for the Development of Evidence-based Patient Information) [37]. Furthermore, the material was designed to address the competence areas mentioned in the mandatory framework for digital education and digital literacy in schools with a special focus on the competence areas: (1) searching, processing, and storing; (2) problem solving and acting; and (3) analyzing and reflecting [32,33]. The e-learning course consists of 8 mandatory modules, 3 optional modules, and a final test. Internal analyses show that it takes about 4 minutes to complete the shortest module and 18 minutes to complete the longest module. The entire course can be completed in about 2 hours. Table 1 shows the length of the e-learning course modules according to internal analyses. Figure 1 shows a screenshot of the e-learning portal Gesundweiser.de. Figure 2 provides an overview of the e-learning course modules. After finishing the e-learning course, participants answered all the measures again (postmeasurement). At the end of the study, participants were thanked for their participation and received their online shop voucher.

**Table 1:** Length of the e-learning course modules according to internal analyses.

Course module	Length (min)
Mandatory module 1	14
Mandatory module 2	18
Optional module 1	6
Mandatory module 3	6
Mandatory module 4	9
Optional module 2	6
Mandatory module 5	14
Mandatory module 6	12
Mandatory module 7	9
Optional module 3	4
Mandatory module 8	11
Final test	11
Complete course	120

### Measures

#### Health Literacy

To assess health literacy, the Health Literacy for School-Age Children Instrument was used [39,40]. This 10-item instrument comprises 5 subscales assessing competencies in the fields of theoretical knowledge (2 items: eg, “I am confident that I have good information about health”), practical knowledge (2 items: eg, “I am confident that when necessary I find health-related information that is easy for me to understand”), critical thinking (2 items: eg, “I am confident that I can usually figure out if some health-related information is right or wrong”), self-awareness (2 items: eg, “I am confident that I can give reasons for choices I make regarding my health”), and citizenship (2 items: eg, “I am confident that I can judge how my own actions affect the surrounding natural environment”). Participants rated all items on scales ranging from 1 (strongly disagree) to 4 (strongly agree). For each subscale, a total score was generated by calculating the mean.

#### Digital Health Literacy

To assess digital health literacy, 3 of the 7 subscales of the Digital Health Literacy Instrument were used [11,20]. The 3 subscales assessed competencies in the fields of information searching (3 items: eg, “When you search the internet for information on health, how easy or difficult is it for you to make a choice from all the information you find?”), evaluating reliability (3 items: eg, “When you search the internet for information on health, how easy or difficult is it for you to decide whether the information is reliable or not?”), and determining relevance (3 items: eg, “When you search the internet for information on health, how easy or difficult is it for you to decide if the information you found is applicable to you?”). Participants rated all items on scales ranging from 1 (very hard) to 4 (very easy). For each subscale, a total score was generated by calculating the mean.

#### Subjective (Digital) Health Literacy Knowledge

To assess subjective knowledge in the domain of (digital) health literacy, participants indicated their agreement with 5 items (eg, “I can explain which content-related characteristics are indicative of reliable health information on the internet)”. The items focused on content covered throughout the e-learning course. Participants rated all items on scales ranging from 1 (strongly disagree) to 6 (strongly agree). A total score was generated by calculating the mean.

#### Objective (Digital) Health Literacy Knowledge

To assess objective knowledge in the domain of (digital) health literacy, participants answered 15 multiple-choice questions, such as “What does the phrase ‘evidence-based’ mean?” with response options (1) reviewed by experts, (2) based on scientific evidence and proof, (3) based on personal views and experiences, and (4) rated as helpful by a certain number of users. The multiple-choice questions focused on content covered throughout the e-learning course. Depending on the multiple-choice question, 1 to 4 of the responses were correct. For each correctly answered multiple-choice question, participants received 1 point. A total score was generated by adding up all points (minimum=0; maximum=15). The original data set contains further variables that have not been described because they exceed the scope of this study.

### Statistical Analysis

For all analyses, the statistical software JASP, version 0.16.1 (University of Amsterdam), was used [41]. To test the hypotheses, Bayesian paired-samples *t* tests (1-sided) were conducted with the following specifications: alternative hypothesis (measure 1 < measure 2), Bayes factor (BF_10_), test (student), missing values (exclude cases per dependent variable), and prior (default Cauchy scale=0.707). These specifications imply that the results will report Bayes factors in favor of the alternative hypotheses (measure 1 < measure 2). Following a commonly used classification scheme, Bayes factors above 1 will be interpreted as anecdotal (1-3), moderate (3-10), strong (10-30), very strong (30-100), or extreme (>100) evidence for the alternative hypothesis compared to the null hypothesis in light of the observed data [42]. Bayes factors below 1 will be interpreted as evidence for the null hypothesis. For all analyses, a Bayes factor robustness check is provided. The robustness check “provides an assessment of the robustness of the Bayes factor under different prior specifications: if the qualitative conclusions do not change across a range of different plausible prior distributions, this indicates that the analysis is relatively robust” [43]. Further information on the interpretation of Bayes factors in medical contexts and nontechnical introductions to Bayesian inference with JASP can be found elsewhere [42-44].

## Results

### Sample Characteristics

A total of 340 participants completed the study; 17 participants were excluded from data analysis because of data collection problems or because they did not finish the e-learning course. Therefore, the final sample contained 323 (188 females, 132 males, 3 diverse) participants from all German states with an average age of 17.88 (SD 1.22) years. Table 2 shows the sample distribution by state, type of school, and grade.

**Table 2:** Sample distribution by state, type of school, and grade.

Characteristic			Sample, n
**State**			
	Baden-Württemberg	30	
	Bayern	41	
	Berlin	11	
	Brandenburg	6	
	Bremen	1	
	Hamburg	16	
	Hessen	34	
	Mecklenburg-Vorpommern	7	
	Niedersachsen	33	
	Nordrhein-Westfalen	78	
	Rheinland-Pfalz	12	
	Saarland	6	
	Sachsen	22	
	Sachsen-Anhalt	7	
	Schleswig-Holstein	7	
	Thüringen	12	
**Type of school**			
	Hauptschule	1	
	Realschule	23	
	Sekundarschule	2	
	Gesamtschule	37	
	Gymnasien	134	
	Berufsschule	78	
	Berufsfachschule	24	
	Fachoberschule	15	
	Other	9	
**Grade**			
	8th	0	
	9th	7	
	10th	45	
	11th	57	
	12th	100	
	13th	47	
	Other	67	

### Findings

Table 3 provides descriptive statistics of the premeasurements and postmeasurements.

**Table 3:** Descriptive statistics of the premeasurements and postmeasurements^a^.

Measure			Mean (SD)		SE		95% Credible interval
**Health literacy**							
	Theoretical knowledge (pre)	2.810 (0.542)		0.030		2.750-2.869	
	Theoretical knowledge (post)	3.008 (0.510)		0.028		2.952-3.064	
	Practical knowledge (pre)	3.017 (0.598)		0.033		2.952-3.082	
	Practical knowledge (post)	3.207 (0.526)		0.029		3.150-3.265	
	Critical thinking (pre)	2.759 (0.565)		0.031		2.697-2.820	
	Critical thinking (post)	3.107 (0.587)		0.033		3.043-3.171	
	Self-awareness (pre)	2.856 (0.583)		0.032		2.792-2.920	
	Self-awareness (post)	3.088 (0.523)		0.029		3.031-3.146	
	Citizenship (pre)	2.850 (0.567)		0.032		2.788-2.912	
	Citizenship (post)	3.053 (0.543)		0.030		2.993-3.112	
**Digital health literacy**							
	Information searching (pre)	2.716 (0.607)		0.034		2.650-2.783	
	Information searching (post)	2.807 (0.566)		0.031		2.745-2.869	
	Evaluating reliability (pre)	2.522 (0.603)		0.034		2.456-2.588	
	Evaluating reliability (post)	2.841 (0.605)		0.034		2.775-2.907	
	Determining relevance (pre)	2.651 (0.583)		0.032		2.587-2.715	
	Determining relevance (post)	2.853 (0.557)		0.031		2.792-2.914	
**(Digital) health literacy knowledge**							
	Subjective (pre)	3.510 (1.068)		0.059		3.393-3.627	
	Subjective (post)	5.202 (0.708)		0.039		5.124-5.279	
	Objective (pre)	5.842 (2.870)		0.160		5.528-6.156	
	Objective (post)	11.022 (2.221)		0.124		10.779-11.265	

^a^Health literacy measures ranged from 1 (low score) to 4 (high score); digital health literacy measures ranged from 1 (low score) to 4 (high score); subjective (digital) health literacy knowledge ranged from 1 (low score) to 6 (high score); and objective (digital) health literacy knowledge ranged from 0 (low score) to 15 (high score).

#### Health Literacy

**Figure 3:**
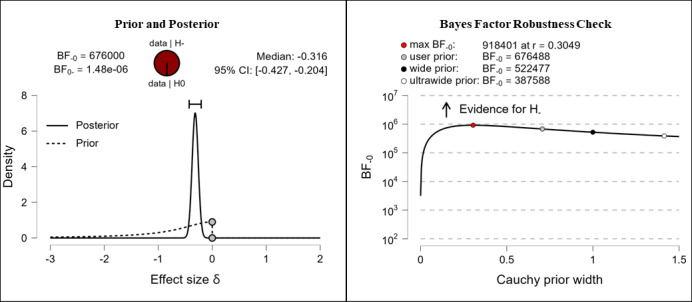
Health literacy: theoretical knowledge. BF: Bayes factor.

**Figure 4:**
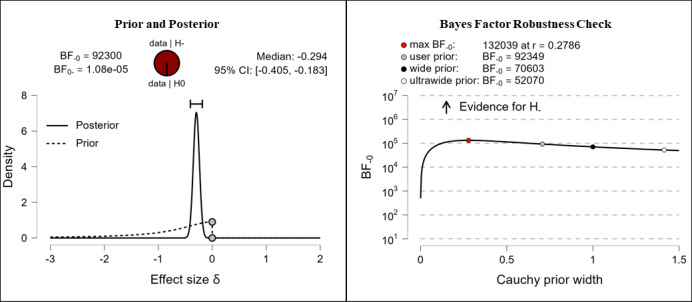
Health literacy: practical knowledge. BF: Bayes factor.

**Figure 5:**
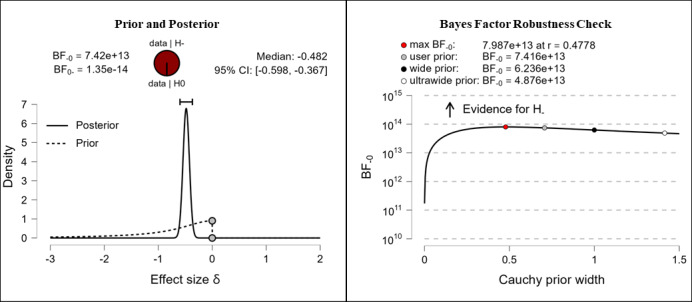
Health literacy: critical thinking. BF: Bayes factor.

**Figure 6:**
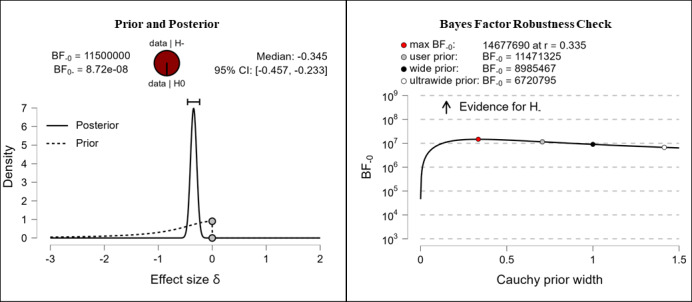
Health literacy: self-awareness. BF: Bayes factor.

**Figure 7:**
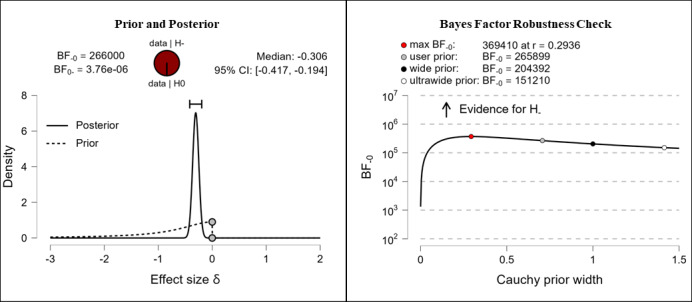
Health literacy: citizenship. BF: Bayes factor.

It was hypothesized that participants’ health literacy levels would be higher after completing the e-learning course than they were before completing the e-learning course. The results show extreme evidence for the hypothesis. After completing the e-learning course, participants reported higher competencies in the fields of theoretical knowledge (extreme evidence, BF_–0_=676,000; δ=–0.316), practical knowledge (extreme evidence, BF_–0_=92,300; δ=–0.294), critical thinking (extreme evidence, BF_–0_=7.42e+13; δ=–0.482), self-awareness (extreme evidence, BF_–0_=11,500,000; δ=–0.345), and citizenship (extreme evidence, BF_–0_=266,000; δ=–0.306). The corresponding prior and posterior distribution plots, effect sizes, and Bayes factor robustness checks are shown in Figure 3 (theoretical knowledge), Figure 4 (practical knowledge), Figure 5 (critical thinking), Figure 6 (self-awareness), and Figure 7 (citizenship).

#### Digital Health Literacy

**Figure 8:**
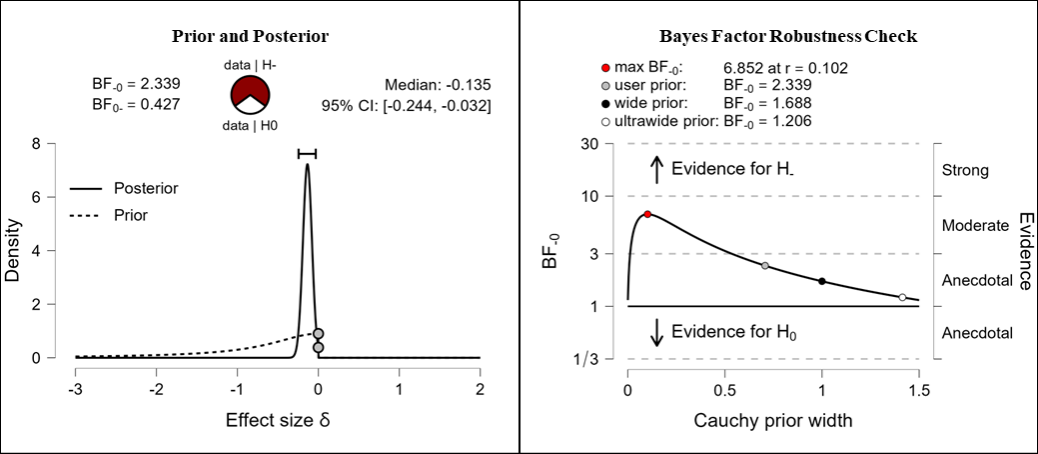
Digital health literacy: information searching. BF: Bayes factor.

**Figure 9:**
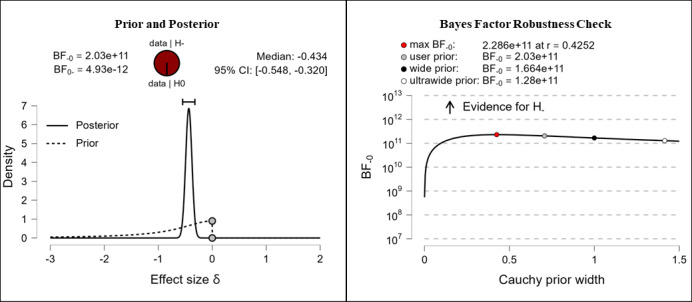
Digital health literacy: evaluating reliability. BF: Bayes factor.

**Figure 10:**
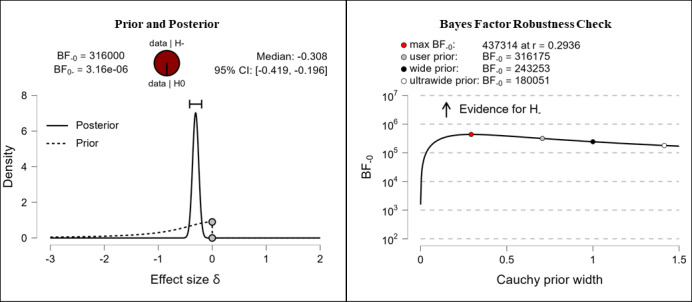
Digital health literacy: determining relevance. BF: Bayes factor.

It was hypothesized that participants’ digital health literacy levels would be higher after completing the e-learning course than they were before completing the e-learning course. Depending on the measured domains, the results vary from anecdotal to extreme evidence for the hypothesis. After completing the e-learning course, participants reported higher competencies in the fields of information searching (anecdotal evidence, BF_–0_=2.339; δ=–0.135), evaluating reliability (extreme evidence, BF_–0_=2.03e+11; δ=–0.434), and determining relevance (extreme evidence, BF_–0_=316,000; δ=–0.308). The corresponding prior and posterior distribution plots, effect sizes, and Bayes factor robustness checks are shown in Figure 8 (information searching), Figure 9 (evaluating reliability), and Figure 10 (determining relevance).

#### Subjective (Digital) Health Literacy Knowledge

**Figure 11:**
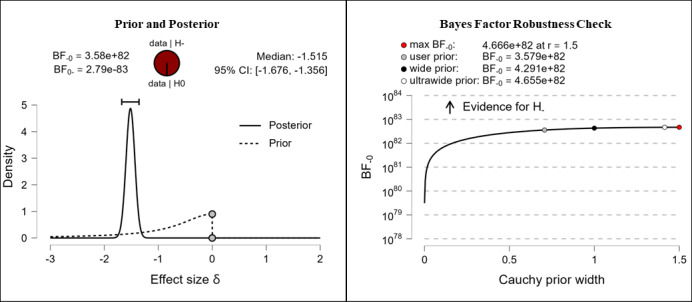
(Digital) health literacy knowledge: subjective. BF: Bayes factor.

It was hypothesized that participants’ subjective knowledge in the domain of (digital) health literacy would be higher after completing the e-learning course than it was before completing the e-learning course. The results show extreme evidence (BF_–0_=3.58e+82; δ=–1.515) for the hypothesis. The corresponding prior and posterior distribution plot, effect size, and Bayes factor robustness check are shown in Figure 11.

#### Objective (Digital) Health Literacy Knowledge

**Figure 12:**
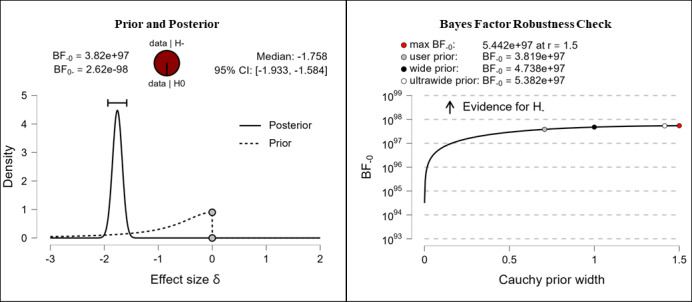
(Digital) health literacy knowledge: objective. BF: Bayes factor.

It was hypothesized that participants’ objective knowledge in the domain of (digital) health literacy would be higher after completing the e-learning course than it was before completing the e-learning course. The results show extreme evidence (BF_–0_=3.82e+97; δ=–1.758) for the hypothesis. The corresponding prior and posterior distribution plot, effect size, and Bayes factor robustness check are shown in Figure 12.

## Discussion

The goal of this study was to evaluate a newly developed e-learning course available on the e-learning platform Gesundweiser.de and its potential to promote (digital) health literacy in school-age children. It was hypothesized that participants’ health literacy (hypothesis 1) and digital health literacy levels (hypothesis 2) would be higher after completing the e-learning course than they were before completing the e-learning course. Furthermore, it was hypothesized that participants’ subjective (hypothesis 3) and objective knowledge (hypothesis 4) in the domain of (digital) health literacy would be higher after completing the e-learning course than it was before completing the e-learning course. The results support all 4 hypotheses. After completing the e-learning course, participants achieved higher health literacy levels. More specifically, they reached higher competency levels in the domains of theoretical knowledge, practical knowledge, critical thinking, self-awareness, and citizenship. Furthermore, participants achieved higher digital health literacy levels. More specifically, they reached higher competency levels in the domains of information searching, evaluating reliability, and determining relevance. Moreover, participants demonstrated higher subjective and objective knowledge in the domain of (digital) health literacy after completing the e-learning course.

There are several reasons why these results are encouraging, and 2 of them seem especially important. First, experts have long argued that (digital) health literacy should be taught to school-age children [24-28]. At the same time, however, there are not many German-language interventions available to promote (digital) health literacy that have been scientifically evaluated and proven to work. The e-learning platform Gesundweiser.de closes this gap by providing a scientifically evaluated e-learning course for school-age children that is freely available for pupils, parents, teachers, and all other interested parties. Second, experts have argued that the promotion of (digital) health literacy should be an integral part of the school curriculum [29,30]. However, promoting (digital) health literacy within the German school system is difficult because (digital) health education is not a mandatory school subject in all German states. Therefore, experts suggest that (digital) health literacy could be addressed as part of the mandatory framework for digital education and digital literacy in schools, which was developed by the German Conference on Education Ministries and Cultural Affairs (Kultusministerkonferenz) [32,33]. Because the presented e-learning course was designed to improve (digital) health literacy and at the same time to teach skills specified in the mandatory framework for digital education and digital literacy in schools, it provides an easy way for schools and teachers from all German states to integrate (digital) health literacy education into their school curriculums and lessons.

Even though the results of this study show that the e-learning course available in the e-learning platform Gesundweiser.de has the potential to promote (digital) health literacy in school-age children, there are limitations to the generalizability of the results. Three limitations seem especially important. The first limitation concerns the age of the study participants. All study participants were aged 16 to 20 years. Because previous research has shown that age might influence the suggestibility to misinformation, source monitoring, and digital literacy, the results of this study may not be generalized to younger age groups [45-47]. Therefore, future research should replicate this study with study participants younger than 16 years to explore whether the e-learning course can also promote (digital) health literacy in younger age groups.

The second limitation concerns the methodological approach that was chosen. This study employed a pre-post measurement study design. This means that study participants answered all measures both before and after completing the e-learning course. One of the main advantages of this methodological approach is that it can reduce random noise. In some circumstances, however, this methodological approach might reveal the aim of the study to the participants. After completing questionnaires about their (digital) health literacy and answering knowledge questions about (digital) health literacy, participants might have guessed that the study was designed to test whether the provided e-learning course has the potential to improve (digital) health literacy. This, in turn, might have induced a demand effect that influenced participants’ evaluations and learning motivation [48]. Therefore, future studies should test the rationale of this study with a different methodological approach. For example, a between-subject experimental design could be chosen in which participants are randomly assigned to an experimental or control group and answer the dependent measures just once at the end of the study.

The third limitation concerns the setting in which participants could complete the e-learning course. To simulate a homework exercise, participants were given 7 days to complete the course. During this 7-day period, participants could use any device to complete the course and they could start and pause the course as often as they liked. Simulating a homework exercise by giving participants the opportunity to complete the course within 7 days has the advantage that it increases ecological validity. However, it also creates methodological disadvantages. It cannot be guaranteed, for example, that participants completed the course without any help from parents or friends. Furthermore, learning results might be influenced by the number of times participants started and paused the course and by whether participants completed the optional modules. Following the principle of data parsimony, data collection focused on the variables that were most relevant for hypothesis testing and no data were collected regarding the number of times participants started and paused the course and whether participants completed the optional modules. To ensure that participants complete the course without any external help and to investigate the effects of completing the optional modules and pausing and restarting the course, future studies could repeat this study in a laboratory setting and control for the described variables.

## Abbreviations

BF: Bayes factor
